# Intraoperative frozen section examination for penile cancer surgery: a systematic review

**DOI:** 10.1038/s41443-025-01024-7

**Published:** 2025-02-11

**Authors:** Mohammad Z. Yunis, Karl H. Pang, Asif Muneer, Hussain M. Alnajjar

**Affiliations:** 1 King’s College London Medical School, London, UK; 2 Institute of Andrology, Department of Urology, University College London Hospitals NHS Foundation Trust, London, UK; 3 Division of Surgery and Interventional Science, University College London, London, UK; 4 Department of Surgical Biotechnology, University College London, London, UK; 5 NIHR Biomedical Research Centre, University College London Hospitals NHS Foundation Trust, London, UK

**Keywords:** Urogenital diseases, Diagnosis

## Abstract

Penile cancer (PeCa) is rare but aggressive and life changing. Penile-preserving surgery (PPS) allows length preservation for sexual activity and normal voiding. Intraoperative frozen section examination (FSE) of resection margins helps to decide on how much penile tissue is excised. Oncological outcomes and diagnostic accuracy of FSE to date, however, are not well documented. The objective of this systematic review was to evaluate the efficacy of FSE in the treatment of PeCa and its impact on oncological outcomes. A systematic review was conducted with reference to the PRISMA statement. Studies published from 2009 to 2024 were identified through a search conducted between 1975 and 2024. The search yielded 7 studies involving 574 patients. Intraoperative FSE had a high percentage of accuracy, with a mean accuracy of 95.4% and a range of 92.9–99.4%. The mean values of sensitivity, specificity, positive predictive value, and negative predictive values were 71.4%, 99.9%, 98.8%, and 96.5%, respectively. Functional outcomes with PPS were encouraging, especially in terms of sexual function. The average local recurrence rate was 7.9%. There is a paucity of data on PeCa FSE in the literature. However, it appears that FSE is accurate and can be helpful in guiding surgeons intraoperatively when performing PPS.

## Introduction

Penile cancer (PeCa) is rare and commonly occurs in the 6th and 7th decades of life [1]. The age-standardised incidence of PeCa was 0.80 per 100,000 men in the world with a total of 37,699 incidences, according to the 2022 Global Cancer Observatory (GLOBOCAN) [2]. According to a population-based observational study, the 5-year survival rate of PeCa between 2002 and 2007 is approximately 70%. Verhoeven et al. showed that this figure does not show statistically significant changes in medically advanced countries like in the European Union [3].

Histopathological analysis plays a crucial role in the diagnosis, risk stratification, assessment of depth of invasion and resection margins of PeCa. Squamous cell carcinoma (SCC) accounts for 95% of PeCa with usual type SCC exhibiting the most common subtype [4]. Historically, a 20 mm macroscopic resection margin was required but with the use of intraoperative frozen section examination (FSE), it allows further resection of a positive margin during the same operation and be conservative with macroscopic margins [5, 6]. Penile-preserving surgery (PPS) preserves penile length, urinary function and sexual function, allowing for standing voiding while reducing psychological impact and avoiding emasculating surgery [7, 8]. The European Association of Urology (EAU) and the American Society of Clinical Oncology (ASCO) recommend that standard excision should encompass a margin of clinically normal-appearing skin around the tumour and any surrounding erythema to achieve complete removal with histologically negative margins [9].

FSE has an important role in determining margin status intraoperatively however, it is not routinely implemented in diagnostic testing because several lesions have well-differentiated squamous cell growth that can mimic non-neoplastic lesions and conversely some hyperplastic lesions can have a pseudoepitheliomatous appearance [10–12]. Patients must be informed of the negative implications of FSE, when there is discordance between FSE and final histology, patients may develop early local recurrence. There is a lack of data on FSE accuracy in the management of PeCa in the literature. In this systematic review, we aim to provide the accuracy of FSE surgical margins and its outcomes in the context of PeCa excisional surgery, alongside a narrative review of the available evidence.

## Methods and materials

### Search strategy

The systematic review was conducted following the PRISMA 2020 reporting guidelines (Supplementary Table 1) [13], and a search was performed on the 26th of January 2023. An updated search was performed on the 12th of September 2024. The review was registered with PROSPERO (ID: 387370). The search strategy was developed by two investigators (MZY and KHP) and is shown in the Supplementary Table 2 for each source. The search strategy was written for PubMed but was translated using each database’s syntax and search fields. Boolean operators “AND” and “OR” were respectively used to include results with both texts and include results with one or the other.

We searched the following databases: PubMed, Cochrane CENTRAL and OVID (Medline and Embase). The retrieved literature from these databases was scarce, therefore we identified other studies by searching on Google Scholar.

All database records were exported to EndNote 20 where duplication, title, and abstract screening occurred [14]. Full-text articles were screened when eligible and reasons for acceptance or rejection were recorded in an Excel spreadsheet (Microsoft Excel version 16).

### Eligibility criteria

Original articles reporting on men with penile lesions affecting any area of the penis were included, and surgical inclusion criteria were excisional surgery of the lesion with the use of intraoperative FSE for curative intent of the lesion, including PPS, partial and radical penectomy. Studies with insufficient data on the concordance of intraoperative FSE with paraffin histology were excluded. Other exclusion criteria were reviews, case reports, editorials, letters, articles not in English and congress abstracts. The population (P), intervention (I), comparison (C), outcome (O), and studies (S) framework used was:

P: patients with PeCa

I: excisional margin FSE

C: diagnostic paraffin section histology

O: diagnostic testing accuracy (DTA) of FSE (e.g., sensitivity, specificity, positive predictive value (PPV), negative predictive value (NPV)), final margin status, sexual and urinary function, survival, recurrence, metastasis and postoperative complications.

S: randomised and non-randomised trials, retrospective or prospective cohort studies and case series.

### Study selection

After completion of our search, the titles of all studies included in the search were screened. Following this, abstracts were screened in studies with eligible titles which allowed the author to then review all the relevant full-text articles. Two investigators (MZY and KHP) independently reviewed the full-text confirming eligibility for our systematic review.

### Data extraction – variables

Extracted variables included study characteristics: author, year of publication, population, adjuvant therapy, and surgery type. For studies which included the pathology data, we looked for FSE margins, final surgical margins, tumour grade, tumour stage, nodal stage, and penile intraepithelial neoplasia (PeIN) or carcinoma in situ (CIS). The primary outcome was the concordance of FSE margins when compared with the final paraffin histology reports. The analysis of the concordance of FSE was done by calculating the DTA: sensitivity, specificity, PPV, NPV. This was done by inputting true positive (TP), false positive (FP), false negative (FN), and true negative (TN) results into MedCalc’s diagnostic test evaluator tool [15]. Accuracy of intraoperative FSE is defined as the probability that a patient is correctly classified: TN being negative margins, and TP being positive margins. For secondary outcomes, we looked at final margin status, sexual and urinary function, survival, recurrence, metastasis, and postoperative complications. A meta-analysis was unable to be performed from the extracted data due to the heterogeneity of available studies, so a descriptive analysis was performed instead.

### Quality assessment of individual studies

The risk of bias (RoB) of the included studies was assessed using the Newcastle-Ottawa Scale (NOS) for evaluating the quality of non-randomised cohort studies [16]. However, we utilised the British Medical Journal’s revision of the NOS scale specifically for case series due to the predominance of this study design in the included literature [17]. We looked at three factors which were considered to assess the quality of each of our 7 studies:

1. Selection: exposed cohort representativeness, selection of controls, ascertainment of exposure, and definition of controls
2. Comparability: assessed on the matching of individuals and any other factors
3. Outcome: on assessment method, follow-up time, and adequacy of follow-up

Each study was classified as having high, moderate or low methodological quality based on the points achieved, with 7–9 points deemed high quality, 5–6 points classified as moderate quality, and fewer than 5 points indicating low methodological quality.

## Results

### Study selection

**Fig. 1:**
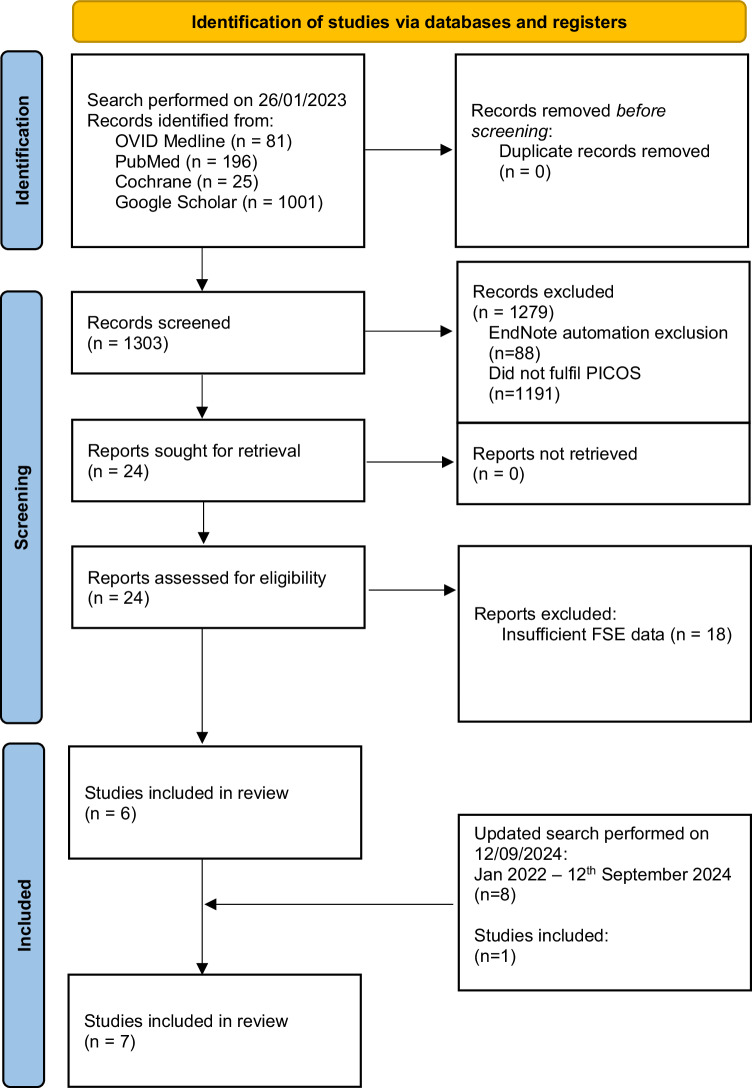
Flow diagram of the search following PRISMA (2020) [13].

Figure 1 describes the study inclusion process in the form of a flow chart following PRISMA (2020) [13]. The initial search on the 26th of January 2023 identified 1303 records. Title and abstract screening were performed, after which 24 studies fulfilled the PICOS criteria, which then underwent further evaluation. From these studies, an additional 18 papers were excluded due to insufficient reporting on the details of FSE, and its concordance with final paraffin histology, which rendered them unsuitable for our systematic review. An updated search was performed on the 12th of September 2024 which retrieved 124 papers, 1 of which was included for analysis. Overall, 7 studies were included, and the extracted data are summarised in Table 1. There were no randomised or non-randomised controlled studies, and all 7 studies included were observational studies. The total number of patients with intraoperative FSE was 574.

**Table 1:** Studies histopathology and outcomes results.

Study	FSE (*n*)	Surgery type (*n*)	Final surgical margins (*n*)	Tumour grade (*n*)	Tumour stage (*n*)	Time to follow-up (months)	Complications (*n*)	Recurrence (*n*)
Li et al. [18]	32	RC (8)	Negative (32)	G1 (21)	pTa (5)	Mean (N/R)	3	3
		WLE (18)	Positive (0)	G2 (1)	pTis (2)	Median (26.5)		
		WLE & CC (6)		G3 (3)	pT1 (23)	Range (2–61)		
				G4 (0)	pT2 (2)			
					pT3 (0)			
Morelli et al. [19]	15	TG (14)	Negative (15)	G1 (6)	pTa (5)	Mean (36)	4	0
		TG & DC (1)	Positive (0)	G2 (8)	pTis (0)	Median (N/R)		
				G3 (1)	pT1 (7)	Range (10–67)		
				G4 (0)	pT2 (4)			
					pT3 (2)			
Danakas et al. [20]	31	PP (21)	Negative (27)	G1 (10)	pTa & pTis (2)	Mean (38)	N/R	5
		TP (10)	Positive (4)	G2 (13)	pT1 (14)	Median (27)		
				G3 (8)	pT2 (7)	Range (1–137)		
				G4 (0)	pT3 (8)			
Ellul et al. [21]	169	PP (77)	Negative (169)	G1 (22)	pTa (1)	Mean (N/R)	N/R	9
		GR (8)	Positive (0)	G2 (57)	pTis (7)	Median (45)		
		PG (6)		G3 (74)	pT1 (50)	Range (6–170)		
		TG (70)		G4 (6)	pT2(70)			
		WLE (9)			pT3 (38)			
					pT4 (1)			
O’Kelly et al. [22]	19	GR (19)	Negative (18)	N/R	N/R	N/R	0	1
			Positive (1)					
Parnham et al. [23]	171	TG (171)	Negative (154)	G1 (7)	pTa (0)	Mean (N/R)	50	19
			Positive (17)	G2 (48)	pTis (0)	Median (41.4)		
				G3 (117)	pT1 (56)	Range (2–155)		
				G4 (0)	pT2 (98)			
					pT3 (21)			
Pang and Yunis et al. [24]	137	GL (72)	Negative (122)	G1 (7)	pTa (0)	Mean (N/R)	N/R	14
		DC (4)	Positive (15)	G2 (31)	pTis (0)	Median (28)		
		GR (5)		G3 (98)	pT1 (27)	Range (9–76)		
		PG (3)		G4 (1)	pT2 (73)			
		PP (41)			pT3 (37)			
		STP (4)						
		TP (5)						

*N/R* not recorded, *PP* partial penectomy, *TP* total penectomy, *STP* subtotal penectomy, *RC* radical circumcision, *CC* circumcision, *WLE* wide local excision, *TG* total glansectomy, *GL* glansectomy, *GR* glans resurfacing, *PG* partial glansectomy, *DC* distal corporectomy.

### Accuracy of frozen section examination margin reports

FSE for PeCa is not routinely used for initial diagnostic purposes. Instead, margins are taken to confirm the complete excision of the tumour. The sensitivity, specificity, PPV, NPV, and accuracy results are summarised in Table 2. In both Li et al. and Morelli et al.’s studies, there was 100% concordance, the PPV and sensitivity calculation cannot be calculated due to 0 patients with positive margin values [18, 19]. All studies showed a high percentage accuracy of intraoperative FSE with a range from 92.9 to 99.4% and a mean accuracy of 95.4%. Additionally, the mean sensitivity, specificity, PPV, and NPV were 71.4%, 99.9%, 98.8%, and 96.5%, respectively [18–24].

**Table 2:** Accuracy of reports comparing FSE of margins results with final histopathology.

Study	Year	FSE (*n*)	Atypical	TP	FP	FN	TN	Sensitivity% (95% CI)	Specificity% (95% CI)	PPV% (95% CI)	NPV (95% CI)	Accuracy%
Li et al. [18]	2011	32	0	0	0	0	32	N/R	100	N/R	100	N/R
									(89.1–100)		(89.1–100)	
Morelli et al. [19]	2009	15	0	0	0	0	15	N/R	100	N/R	100	N/R
									(78.2–100)		(78.2–100)	
Danakas et al. [20]	2018	31	3	3	0	2	23	60	100	100	92	92.9
								(14.7–94.7)	(85.2–100)	(29.2–100)	(79.7–97.1)	(76.5–99.1)
Ellul et al. [21]	2020	169	1	20	1	0	147	100	99.3	95.2	100	99.4
								(83.2–100)	(96.3–100)	(73.9–99.3)	(97.5–100)	(96.7–100)
O’Kelly et al. [22]	2017	19	0	0	0	1	18	N/R	100	N/R	94.7	94.7
									(81.5–100)		(94.7–94.7)	(74.0–99.9)
Parnham et al. [23]	2018	171	0	10	0	7	154	58.8	100	100	95.7	96
								(32.9–81.6)	(97.6–100)	(69.2–100)	(92.6–97.5)	(91.8–98.3)
Pang and Yunis et al. [24]	2024	137	3	16	0	8	110	66.7	100	100	93.2	94
								(44.7–84.4)	(96.7–100)	(79.4–100)	(88.7–96.0)	(88.6–97.4)

Analysis performed with MedCalc [15].

*FSE* frozen section assessment, *TP* true positive, *FP* false positive, *FN* false negative, *TN* true negative, *CI* confidence interval, *PPV* positive predictive value, *NPV* negative predictive value, *N/R* not recorded.

### Indications of frozen section examination in penile cancer surgery

Radical penectomy and total penectomy can have intraoperative FSE indications for ensuring that the excision margin is clear of PeCa. Intraoperative FSE during total penectomy is performed at the discretion of the surgeon or in line with the departmental policy. Danakas et al. showed the benefit of FSE during total penectomy with 4 cases showing negative conversion of margins with initial positive or atypical FSE. However, they concluded that there was not a significant impact on final surgical margin status nor long-term oncologic outcomes with the use of FSE [20].

PPS can be performed in cases where it is possible to completely excise the tumour and maintain a functional penile length. Studies utilising partial penectomy, radical circumcision, wide local excision (WLE), glansectomy and glans resurfacing were reviewed. Parnham et al. presented 171/177 patients from February 2005 to January 2016 who underwent glansectomy and split-thickness skin graft (STSG) with intraoperative FSE. They described biopsies of the complete circumference of the urethra and of each corporal tip which was sent for FSE. These improved outcomes in at least 10 patients who had further resection following a positive FSE result [23]. A further 15 cases of glansectomy were shown to prove 100% accuracy [19]. Additionally, FSE can be performed in total glans resurfacing where spongiosal biopsies of the deep margin are taken. O’Kelly et al. presented total glans resurfacing, showing 95% accuracy of FSE where one case had an FN result [22].

### Final margin status and risk of recurrence

Local recurrence (LR) was a secondary outcome measure assessed. One study suggested that LR most likely occurs within 6 months post-operation [18], whereas another suggested that it occurs within 2 years [21]. Two studies provided the median time of recurrence at 8.7 and 10 months [21, 23]. The range of LR in all studies was 0–16%, although patients were not followed up for the same amount of time in the selected reports, the mean risk of patients having a local recurrence was 7.9% across all studies [18–24]. Danakas et al. suggested that patients with positive surgical margins tend to have a higher risk for disease recurrence, compared with those with negative surgical margins [20]. This suggested that the risk of a positive margin is greater when PeCa is removed without the use of intraoperative FSE. Pang and Yunis et al. examined the local recurrence rate in 137 patients undergoing PPS surgery for penile SCC. In this study, the overall LR rate was 10.2%. Among patients with positive FSE margins, the recurrence rate was higher at 25%, compared to 8.5% in those with negative FSE margins [24]. This suggested that positive surgical margins, even when identified and addressed with further resection during surgery, may still carry a higher risk of LR.

### Postoperative complications and patient-reported outcomes

Across 4 studies, where complications were reported, there was an average complication rate of 17.7% [18, 19, 22, 23]. Li et al. presented 3/32 (9.4%) complications where 2 patients had wound dehiscence, and 1 patient had the formation of a local abscess [18]. Whilst Morelli et al. presented a higher rate of complications at 26.7% with 2 partial graft loss’, 1 meatal stenosis, and 1 phimosis [19]. The highest complication rate in one study was 34.5%, where 29 patients had partial graft loss, 5 had complete or near-complete graft loss, 12 underwent regrafting, and 4 reported meatal stenosis [23]. Depending on the type of complication, the patients underwent further surgery, required pharmacological intervention to manage the complication or had conservative management. However, one study reported 0% complications following 19 total glans resurfacing operations [22]. Additionally, 3 studies assessed changes in sexual function. One study showed that 24.1% of patients maintained poor sexual function, and the other 75.9% had no change after surgery [18]. Whereas another study presented an 81% improvement in sexual function [19]. The final study maintained sexual functionality in all patients [22].

Two studies from this review analysed metastatic disease following surgery. Pang and Yunis et al. show 24.1% of patients with regional or distant metastasis post-surgery with a median follow-up to metastasis of 5 months [24]. Li et al.’s cohort of patients had 5 (15.6%) with lymph node metastases, none of whom had adjuvant therapy before or after surgery. All of their patients reported satisfactory urination [18].

### Validity assessment

The RoB was evaluated in each of the papers, results are outlined in Supplementary Table 3. Danakas et al. received a high-quality rating, whilst the other 6 studies received a moderate-quality rating [18–24]. Confounders were identified in each of the studies, which included tumour stage, tumour grade, nodal stage, quality of excision (surgeon preference or case complexity), and prior therapy, however, Danakas et al. showed no statistically significant difference in the histopathological characteristics of the patients [20].

## Discussion

The accuracy of FSE is an important factor to consider when deciding whether its use is valuable intraoperatively. FSE is labour-intensive due to the requirement of available dedicated expert histopathologists, pathology technicians and porters. It is also time-consuming and lengthens the surgery. Some studies have indicated that it is not 100% accurate in its assessment of margins [25], therefore, it is important to weigh the benefits and risks of intraoperative FSE. With the use of intraoperative FSE, it is possible to operate with less macroscopic surgical clearance and re-resect the margins under the same general anaesthetic in the event of a positive margin. This approach reduces the need to reoperate in the future and hence reduces the risk of LR [26]. Our results showed that FSE is accurate in the assessment of penile surgical margins, and it is important to note that there are multiple other studies which advocate the use of FSE during PeCa to attain negative margins [27, 28]. Follow-up for local PeCa recurrence should be done every 3 months for the first 2 years and then every 6 months until at least 5 years post-operatively for PPS [9]. FSE may reduce the risk of recurrence which may be due to the better attainment of negative margins. A study in 2004 by Pietrzak et al. showed that intraoperative FSE can maintain penile length, shape and function without reducing oncological control, and 2 patients who did not have FSE had early complications [29]. Preserved penile form and functional outcomes have been associated with PPS. Partial or total penectomy is associated with major sexual and functional compromise. It is also associated with substantial psychological deficits in more than 50% of patients [30]. Emerging technologies such as optical biopsy with in vivo confocal laser endomicroscopy (CLE) offer a potential alternative or adjunct to FSE. CLE allows for instantaneous real-time, non-invasive visualisation of tissue microstructure, potentially aiding in margin assessment and reducing the need for excisional biopsies [31].

### Limitations

Whilst FSE can be used to confirm negative margins in partial or total penectomy, it is also advantageous in PPS [25]. Our results reflect the positive outcomes of several types of PPS but are also a limitation due to the lack of continuity in the types of surgeries included and the retrospective nature of the studies. As there have not been many published reports on the accuracy of FSE, we have pooled all types of PPS and radical penectomy. More data is required on this topic before stronger evidence, including a meta-analysis, can be performed. Furthermore, there are limited reports comparing FSE to control, and this may be due to ethical considerations and different departmental protocols for operating. Other limitations of the studies included a small sample size as there have not been any large prospective studies to date. Additionally, there was a scope of selection bias as the FSE cases were not randomised. Another limitation of our study was the atypical results in FSE, all of which had further excision of tissue [20, 24], but our method of analysis with MedCalc did not have an input for atypical results [15]. Although atypical results are rare, it does put a patient at increased risk of having further tissue excised leading to a short penile stump which can result in sexual dysfunction, negative mental implications and voiding issues, when the margins are already negative. Additionally, every study included was subject to confounding factors, the largest being different surgery types leading to different outcomes. More reports are required before a review can be done for each specific surgical procedure for the treatment of PeCa.

### Clinical implications

Our results reflect that FSE use in clinical practice is safe and practical, in cases where the resection margins are uncertain. It can be used in line with PPS techniques to give patients greater functional outcomes, whilst maintaining good oncological control with a reduced risk of recurrence, all without the need for a further surgical and anaesthetic episode. Both the surgeon and histopathologist have crucial roles in excising oncologically safe and complete margins and giving accurate clinical information, respectively. For best results, close communication between both the surgeon and histopathologist is suggested to be critical for the assessment of frozen section specimens [11]. Future research should not only focus on larger, prospective studies comparing FSE to control groups but also explore the potential of CLE in PeCa management. Investigating the diagnostic accuracy, feasibility, and impact of CLE on surgical decision-making and patient outcomes could reveal its potential to complement or even replace FSE in certain scenarios.

## Conclusions

The evidence involving FSE is still evolving. There are clear advantages of performing FSE, with reports of high accuracy. FSE enables operating surgeons to excise a penile tumour with the confidence of being able to achieve negative surgical margins. As a result, the risk of LR is reduced with improved patient-reported outcomes. However, PeCa is rare and as centralisation of care and multi-institutional collaboration increases there will be further evidence on the accuracy of FSE.

## Supplementary information

Supplemental Material 2

Supplemental Material 3

Sup Table 1 FSE PeCa SR PRISMA checklist REVISED

## Data Availability

All data generated or analysed during this study are included in this published article [and its supplementary information files].
