# Supplementary material for: Intraoperative frozen section examination for penile cancer surgery: a systematic review
Source: Int J Impot Res. 2025 Feb 11;37(9):721–7. doi: 10.1038/s41443-025-01024-7 (PMC12474546; doi:10.1038/s41443-025-01024-7)
Supplement: Supplementary file 1 — Supplemental Material 2 [file 41443_2025_1024_MOESM1_ESM.docx]

**Supplementary Table 2.** Search terms.

| **Database / Registers** | **Search strategy** |
| --- | --- |
| **PubMed** | ((penile cancer) OR (penile conserving surgery) OR penectomy OR glansectomy OR (glans resurfacing) OR (wide local excision) OR Circumcision OR (penile preserving surgery)) AND (frozen section) |
| **OVID Medline (Embase + Medline)** | 1. penile cancer OR (penile conserving surgery) OR penectomy OR glansectomy OR (glans resurfacing) OR (wide local excision) OR Circumcision OR (penile preserving surgery) 2. (frozen section) 3. 1 AND 2 |
| **Cochrane database** | 1. penile cancer OR (penile conserving surgery) OR penectomy OR glansectomy OR (glans resurfacing) OR (wide local excision) OR Circumcision OR (penile preserving surgery) 2. (frozen section) 3. 1 AND 2 |
| **Google Scholar** | ((penile cancer) OR (penile conserving surgery) OR penectomy OR glansectomy OR (glans resurfacing) OR (wide local excision) OR Circumcision OR (penile preserving surgery)) AND (frozen section) |
