# Supplementary material for: Intraoperative frozen section examination for penile cancer surgery: a systematic review
Source: Int J Impot Res. 2025 Feb 11;37(9):721–7. doi: 10.1038/s41443-025-01024-7 (PMC12474546; doi:10.1038/s41443-025-01024-7)
Supplement: Supplementary file 2 — Supplemental Material 3 [file 41443_2025_1024_MOESM2_ESM.docx]

**Supplementary Table 3.** Risk of bias assessment. Newcastle Ottawa Quality Assessment Scale Criteria [16].

| **Study** | **Selection** | | | **Comparability** | | | **Outcome** | | | **Total** |
| --- | --- | --- | --- | --- | --- | --- | --- | --- | --- | --- |
|  | **Representativeness** | **Selection of controls** | **Ascertainment of exposure** | **Definition of controls** | **Comparability of cases on the basis of design or analysis** | **Cases with comparability on any other factors** | **Assessment of outcome** | **Long enough follow-up** | **Adequacy of follow-up** |  |
| Li et al [18] | * | * | * |  |  |  | * | * | * | 6 |
| Morelli et al [19] | * |  | * |  |  |  | * | * | * | 5 |
| Danakas et al [20] | * | * | * | * |  |  | * | * | * | 7 |
| Ellul et al [21] | * |  | * |  |  |  | * | * | * | 5 |
| O’Kelly et al [22] | * |  | * |  |  |  | * | * | * | 5 |
| Parnham et al [23] | * |  | * |  |  |  | * | * | * | 5 |
| Pang and Yunis et al [24] | * |  | * |  | * |  | * | * | * | 6 |
